# Supplementary material for: A single-progenitor model as the unifying paradigm of epidermal and esophageal epithelial maintenance in mice
Source: Nat Commun. 2020 Mar 18;11:1429. doi: 10.1038/s41467-020-15258-0 (PMC7080751; doi:10.1038/s41467-020-15258-0)
Supplement: Supplementary file 1 — Description of Additional Supplementary Files [file 41467_2020_15258_MOESM1_ESM.pdf]

Title: Supplementary Data 1:
Description: Frequency of Histone-GFP retaining cells at 18 day time point (back skin 14 days) in histone-GFP dilution assay in R26M2rtTA/TetO-H2BGFP mice. CD45+ cells are leukocytes.

Title: Supplementary Data 2:
Description: Quantification of Histone-GFP dilution in esophagus and epidermis of *R26M2rtTA/TetO-H2BGFP* mice.

Title: Supplementary Data 3:
Description: Tests of unimodality of Histone-GFP distribution in Histone-GFP dilution in esophagus and epidermis of *R26M2rtTA/TetO-H2BGFP* mice.

Title: Supplementary Data 4:
Description: Parameter values used in simulations of model fits to experimental data and goodness of fit statistics.

Title: Supplementary Data 5:
Description: Lineage tracing data from *Lrig1-eGFP-cre*^{ERT} *R26*^{flConfetti} mice. Labelled clone sizes in murine esophageal epithelium at different times post-induction (number of basal-layer cells and total cells per labelled clone).

Title: Supplementary Movie 1.
Description: Orthogonal views of a rendered confocal Z stack of a typical esophageal epithelium wholemount from a *Rosa26*^{M2rtTA} *TetO-H2BGFP* mouse immediately after doxycycline induction (0 time point). Main panel is top down (x-y) view, small panels show side (x-z and y-z) views. Green: H2B-GFP; blue: DAPI. The video transits from deepest, basal plane to outermost suprabasal layers.

Title: Supplementary Movie 2.
Description: Orthogonal views of a rendered confocal Z stack of a typical ear epidermis wholemount from a *Rosa26*^{M2rtTA} *TetO-H2BGFP* mouse immediately after doxycycline induction (0 time point). Main panel is top down (x-y) view, small panels show side (x-z and y-z) views. Green: H2B-GFP; blue: DAPI. The video transits from deepest, basal plane to outermost suprabasal layers.

Title: Supplementary Movie 3.
Description: Orthogonal views of a rendered confocal Z stack of a typical tail epidermis wholemount from a *Rosa26*^{M2rtTA} *TetO-H2BGFP* mouse immediately after doxycycline induction (0 time point). Main panel is top down (x-y) view, small panels show side (x-z and y-z) views. Green: H2B-GFP; blue: DAPI; white: KRT14. The video transits from deepest, basal plane to outermost suprabasal layers. A scale plate (most apical, blister-shaped region) is located in the middle of the image, surrounded by deeper interscale regions and hair follicle openings (brightest H2BGFP intensity).

Title: Supplementary Movie 4.
Description: Orthogonal views of a rendered confocal Z stack of a typical dorsum epidermis wholemount from a *Rosa26*^{M2rtTA} *TetO-H2BGFP* mouse immediately after doxycycline induction (0 time point). Main panel is top down (x-y) view, small panels show side (x-z and y-z) views. Green: H2B-GFP. The video transits from deepest, basal plane to outermost suprabasal layers.
