# Supplementary material for: A single-progenitor model as the unifying paradigm of epidermal and esophageal epithelial maintenance in mice
Source: Nat Commun. 2020 Mar 18;11:1429. doi: 10.1038/s41467-020-15258-0 (PMC7080751; doi:10.1038/s41467-020-15258-0)
Supplement: Supplementary file 3 — Reporting Summary [file 41467_2020_15258_MOESM3_ESM.pdf]

nature research

Corresponding author(s): Philip Jones

Last updated by author(s): Feb 01 2020

Reporting Summary

Nature Research wishes to improve the reproducibility of the work that we publish. This form provides structure for consistency and transparency in reporting. For further information on Nature Research policies, see Authors & Referees and the Editorial Policy Checklist .

Statistics

For all statistical analyses, confirm that the following items are present in the figure legend, table legend, main text, or Methods section.

n/a	Confirmed	
☐	☒	The exact sample size (*n*) for each experimental group/condition, given as a discrete number and unit of measurement
☐	☒	A statement on whether measurements were taken from distinct samples or whether the same sample was measured repeatedly
☐	☒	The statistical test(s) used AND whether they are one- or two-sided<br>*Only common tests should be described solely by name; describe more complex techniques in the Methods section.*
☒	☐	A description of all covariates tested
☒	☐	A description of any assumptions or corrections, such as tests of normality and adjustment for multiple comparisons
☐	☒	A full description of the statistical parameters including central tendency (e.g. means) or other basic estimates (e.g. regression coefficient) AND variation (e.g. standard deviation) or associated estimates of uncertainty (e.g. confidence intervals)
☒	☐	For null hypothesis testing, the test statistic (e.g. *F*, *t*, *r*) with confidence intervals, effect sizes, degrees of freedom and *P* value noted<br>*Give P values as exact values whenever suitable.*
☐	☒	For Bayesian analysis, information on the choice of priors and Markov chain Monte Carlo settings
☐	☒	For hierarchical and complex designs, identification of the appropriate level for tests and full reporting of outcomes
☒	☐	Estimates of effect sizes (e.g. Cohen's *d*, Pearson's *r*), indicating how they were calculated

Our web collection on statistics for biologists contains articles on many of the points above.

Software and code

Policy information about availability of computer code

Data collection	Individual-cell H2BGFP intensities were using an object-recognition macro in ImageJ version 1.52p
Data analysis	Model dynamics were simulated using Markovian (Gillespie algorithm) and non-Markovian exact stochastic Monte Carlo methods implemented in Matlab. All code is deposited on GitHub https://github.com/gp10/Piedrafita_etal_SI_code/ .

For manuscripts utilizing custom algorithms or software that are central to the research but not yet described in published literature, software must be made available to editors/reviewers. We strongly encourage code deposition in a community repository (e.g. GitHub). See the Nature Research guidelines for submitting code & software for further information.

Data

Policy information about availability of data

All manuscripts must include a data availability statement. This statement should provide the following information, where applicable:

- Accession codes, unique identifiers, or web links for publicly available datasets
- A list of figures that have associated raw data
- A description of any restrictions on data availability

The authors declare that the experimental data supporting the findings of this study are available within the paper and its supplementary information files.

Field-specific reporting

Please select the one below that is the best fit for your research. If you are not sure, read the appropriate sections before making your selection.

☒ Life sciences ☐ Behavioural & social sciences ☐ Ecological, evolutionary & environmental sciences

Life sciences study design

All studies must disclose on these points even when the disclosure is negative.

ample size	Sample sizes for lineage tracing and histone GFP measurement were determined from previous publications (Doupe et al. Science 2012, Murai et al., Cell Stem Cell, 2018, both cited).
Data exclusions	No data was excluded from the analyses.
Replication	For lineage tracing and Histone measurements, each mouse is an experimental unit, experiments were performed using three or four mice per time point. Findings in each animal were consistent with the whole dataset.
Randomization	Mice of the correct genotype were allocated randomly to experiments and those sampled at each time point were selected randomly.
Blinding	Blinding was not performed as the data are time courses and an experienced observer will know the time point based on the relative clone size or fluorescence intensity.

Reporting for specific materials, systems and methods

We require information from authors about some types of materials, experimental systems and methods used in many studies. Here, indicate whether each material, system or method listed is relevant to your study. If you are not sure if a list item applies to your research, read the appropriate section before selecting a response.

Materials & experimental systems

n/a	Involved in the study	
☐	☒	Antibodies
☒	☐	Eukaryotic cell lines
☒	☐	Palaeontology
☐	☒	Animals and other organisms
☒	☐	Human research participants
☒	☐	Clinical data

Methods

n/a	Involved in the study	
☒	☐	ChIP-seq
☒	☐	Flow cytometry
☒	☐	MRI-based neuroimaging

Antibodies

ntibodies used	Primary antibodies : Lrig1 antibody (R&D Systems, Cat. AF3688, dilution 1:100), ITGA6 antibody (clone GoH3, Biolegend, Cat. B204094 dilution 1:500), Alexa Fluor® 647 anti-CD45 (clone 30-F11, Biolegend, Cat. 103124, dilution 1:200), Keratin 14 antibody (clone Poly19053, Biolegend, Cat. 905301 Dilution 1:1000). Secondary antibodies: Goat or Donkey Alexa Fluor 488/546/555/647 (Molecular Probes).
Validation	Lrig1 antibody (R&D Systems, Cat. AF3688): Immunofluorescence (IF) in mouse validation, this study and https ://www.ncbi.nl m.n ih.gov /pu bmed/19427292. ITGA6 antibody (clone GoH3) IF in mouse,validation in https:// www.ncbi.nlm.nih.gov/pubmed/8673140. anti-CD45 (clone 30-Fll, Biolegend, Cat. 103124), IF in mouse validation in https:// www.nature.com/articles/ncb3400#MOESM38. Krt14 antibody (clone Poly19053, Biolegend, Cat. 905301), IF in mouse, validation in https://www.ncbi.nlm.nih.gov/ pmc/articles/PMC6261459.

Animals and other organisms

Policy information about studies involving animals; ARRIVE guidelines recommended for reporting animal research

aboratory animals	Male and female C57Bl6/N mice maintained on standard chow in independently ventilated cages with environmental enrichment in an SOPF health status animal house were used for this study. Ages ranged for 3 to 18 months.
Vild animals	No wild animals were used in this study. *if*
eld-collected samples	No field-collected samples were used in this study.
Ethics oversight	All experiments were conducted according to the UK Home Office Project Licenses 70/7543, P14FED054 or PF4639B40.

Note that full information on the approval of the study protocol must also be provided in the manuscript.
